# Vigo Insight Monitoring Scale in Schizophrenia (VIMS): validation in a sample of patients with schizophrenia

**DOI:** 10.1192/j.eurpsy.2022.1973

**Published:** 2022-09-01

**Authors:** R. Vazquez Noguerol Mendez

**Affiliations:** HOSPITAL NICOLAS PEÑA, Servicio De PsiquiatrÍa, VIGO, Spain

**Keywords:** insight, scale, schizophrénia

## Abstract

**Introduction:**

Lack of awareness of the disease is one of the most frequent symptoms (<80%) of schizophrenia, and it is accepted to have different aspects: cognitive, related to compliance, specific symptoms, and temporary. The detection of those dimensions of insight affected, allows to select and prioritize the objectives and therapeutic strategies to improve it.

**Objectives:**

To develop a multidimensional scale for monitoring insight in schizophrenia patients

**Methods:**

A scale with 9 insight dimensions has been developed: appreciation of symptoms, acceptance of the cause, clinical and functional repercussions, limitations and level of competence, expected evolution and prognosis, terapeutic, and other factors. risk of decompensation. Each dimension is weighted from 0-4 points, and the result is expressed numerically and graphically. The scale was administered to 60 patients with schizophrenia on three occasions. The initial one by two psychiatrists consecutively, and the third three months after stable treatment. Other clinical and sociodemographic variables were also collected.

**Results:**

In the analysis, reliability, internal consistency, and intra- and interobserver reliability, logical, content, criterion and construct validity were assessed, obtaining satisfactory results in Cronbach’s coefficients and Pearson’s correlation (> 0.7 and > 0.8).

**Conclusions:**

The scale has good reproducibility, validity, sensitivity and utility characteristics, which allow its use in patients with schizophrenia.

**Disclosure:**

No significant relationships.

